# Optimal Lead Time for Dengue Forecast

**DOI:** 10.1371/journal.pntd.0001848

**Published:** 2012-10-18

**Authors:** Yien Ling Hii, Joacim Rocklöv, Stig Wall, Lee Ching Ng, Choon Siang Tang, Nawi Ng

**Affiliations:** 1 Umeå Centre for Global Health Research, Epidemiology and Global Health, Department of Public Health and Clinical Medicine, Umeå University, Umeå, Sweden; 2 Environmental Health Institute, National Environment Agency, Singapore, Singapore; 3 Environment Health Department, National Environment Agency, Singapore, Singapore; Emory University, Kenya

## Abstract

**Background:**

A dengue early warning system aims to prevent a dengue outbreak by providing an accurate prediction of a rise in dengue cases and sufficient time to allow timely decisions and preventive measures to be taken by local authorities. This study seeks to identify the optimal lead time for warning of dengue cases in Singapore given the duration required by a local authority to curb an outbreak.

**Methodology and Findings:**

We developed a Poisson regression model to analyze relative risks of dengue cases as functions of weekly mean temperature and cumulative rainfall with lag times of 1–5 months using spline functions. We examined the duration of vector control and cluster management in dengue clusters > = 10 cases from 2000 to 2010 and used the information as an indicative window of the time required to mitigate an outbreak. Finally, we assessed the gap between forecast and successful control to determine the optimal timing for issuing an early warning in the study area. Our findings show that increasing weekly mean temperature and cumulative rainfall precede risks of increasing dengue cases by 4–20 and 8–20 weeks, respectively. These lag times provided a forecast window of 1–5 months based on the observed weather data. Based on previous vector control operations, the time needed to curb dengue outbreaks ranged from 1–3 months with a median duration of 2 months. Thus, a dengue early warning forecast given 3 months ahead of the onset of a probable epidemic would give local authorities sufficient time to mitigate an outbreak.

**Conclusions:**

Optimal timing of a dengue forecast increases the functional value of an early warning system and enhances cost-effectiveness of vector control operations in response to forecasted risks. We emphasize the importance of considering the forecast-mitigation gaps in respective study areas when developing a dengue forecasting model.

## Author Summary

A dengue early warning system that would provide an accurate forecast could enhance the effectiveness of dengue control, but only if it is given in sufficient time for local authorities to implement those control operations. In this study, we have suggested the optimal timing for issuing a warning of a dengue outbreak in Singapore that will allow authorities adequate time to respond. We first analyzed the relationship between the risk of dengue cases and weather predictors at 1–5 month lag times to gauge the possible lead time for providing an accurate dengue forecast. We then determined the average time needed for local authorities to curb the outbreak of clusters of 10 dengue cases or more using vector control and cluster duration records for the period 2000–2010. Increasing weekly mean temperature and cumulative rainfall preceded a rise in dengue cases up to 5 months with higher risks evident at a lag time of 3–4 months. Local authorities required an average of 2 months with a maximum of 3 months for effective control. Therefore, a dengue early warning given at least 3 months ahead of time would provide sufficient time for local authorities to moderate an outbreak.

## Introduction

Dengue is a viral infection transmitted primarily by *Aedes aegypti* and *Aedes albopictus*. Globally, it is estimated that the disease infects from 50 to 100 million people annually leading to approximately half a million severe dengue cases and 12,500 deaths [1]. The Asia Pacific region shoulders the greatest burden of dengue with about 70% of the 2.5 billion people residing in the region being at risk [2].

Dengue has been identified as a public health challenge in Singapore since the early 1960s but was controlled for more than 2 decades since the 1970's through a comprehensive vector control program. However, the mosquito borne disease re-emerged with increasingly serious outbreaks in the late 1990s [3]. Some of the factors that have contributed to the re-emergence are believed to be 1) low herd immunity in the human population due to decades of low transmission, 2) increase in human population density, 3) increased urbanization which has led to the geographical expansion of the dengue vectors, 4) globalization and increased population movement, and 5) increased fitness of viruses [4], [5]. Singapore experienced an unprecedented outbreak of dengue in the year 2005. Since then, the trend has started to be reversed through an enhanced integrated evidence-based dengue prevention program.

Lack of vaccines or drugs for dengue prevention and treatment has rendered vector surveillance and control the only effective method for reducing disease burden. To combat the recent upsurge in dengue cases, Singapore implemented an active dengue surveillance system alongside continuous dengue risk assessment and monitoring system to detect areas or clusters at risk of dengue outbreaks. The National Environment Agency (NEA) of Singapore receives surveillance data including number of dengue cases, circulating serotypes of the dengue viruses, and larval density. This data is uploaded into a Geographical Information System (GIS) in order to perform real time vector monitoring, dengue risk analysis, and control [6], [7]. A key novel feature is the incorporation of a decision support system focusing on 4 areas – case, virus, and entomological surveillance, and also ecological information such as weather parameters. Surveillance and ecological data are used for temporal and spatial risk stratification, which forms the core of the decision support system and facilitates optimal deployment of resources in time and space [8], [9].

Dengue surveillance could provide early detection of a potential outbreak with higher precision than the model-based forecast, but would offer a much shorter time window for preventive measures [10]. A timely dengue early warning system with accurate forecasting could enhance existing surveillance systems by giving warning weeks or months ahead, thus, allowing a larger window for successful implementation of control.

The United Nations has defined early warning as “*The provision of timely and effective information, through identified institutions, that allows individuals exposed to hazard to take action to avoid or reduce their risk and prepare for effective response*” (International Strategy for Disaster Reduction (ISDR), United Nations (UN), 2004) [11]. The main purpose of a dengue early warning is to provide sufficient time for decision making and effective measures to prevent or moderate an outbreak [12]. In determining the optimal time to issue a warning it is vital to balance the need for accuracy with the need for adequate time to avert or moderate an outbreak.

Studies have shown that climatic factors influence the magnitude of spatiotemporal distribution of dengue cases [13], [14], [15], [16], because of their effects on 1) the life cycle development, population size, biting rates, infective and survival rates of mosquitoes, and 2) the extrinsic incubation period of dengue viruses [17], [18], [19], [20], [21]. The effects of weather on mosquitoes and dengue viruses also influence the length of time lag between exposure to weather and occurrence of dengue cases [22], [23]. This lag time creates a forecast lead time which then provides a window for local authorities to implement measures to prevent or reduce the size of an outbreak. Although there have been several studies in recent years on dengue forecast modeling based on weather parameters, the optimal timing of a warning of a dengue outbreak in the context of response or mitigation by local authority has not yet, to our knowledge, been formally addressed.

### Objectives

This study aims to identify the optimal timing for issuing a dengue early warning that will allow sufficient time for the local authority in Singapore to execute preventive measures to mitigate the potential risks. Our objectives are to 1) establish links between the estimated forecast lead time (lag time) and the time frame required by the local authorities for successful mitigation, 2) analyze time gaps between dengue forecast and successful mitigation, 3) suggest an optimal dengue forecast lead time that provides sufficient time for successful mitigation, and 4) identify possible factors influencing the gap between dengue early warning and mitigation.

## Methods

### Study area

Singapore is an island state nation with an area of about 700 km^2^ and population density of approximately 7000 per km^2^. The nation experiences tropical climate, with temperature between 25°C and 30°C whole year round. The average annual rainfall is 2,200 mm, with the cooler monsoon season from November to January contributing 37% of the precipitations.

### Data

#### Weather data

Daily mean temperature and rainfall were extracted from the National Climatic Data Center, National Oceanic and Atmospheric Administration (NOAA), USA [24]. These data were computed into weekly unit for statistical analysis. During the years 2000–2010, weekly mean temperature ranged from 25.5°C to 30.4°C with average and median temperature about 27.8°C. Weekly cumulative rainfall ranged from 0 to 394.2 mm with median and average rainfall of 30.5 mm and 43 mm, respectively.

#### Dengue cases

Weekly dengue cases from 2000 to 2010 were obtained from the Weekly Infectious Diseases Bulletins of Communicable Diseases Division, Ministry of Health Singapore [25]. According to the Infectious Diseases Act of Singapore, it is mandatory for medical practitioners and laboratories to notify all diagnosed or confirmed cases of dengue to the Ministry of Health within 24 hours.

#### Dengue clusters

Data on dengue clusters and duration of cluster management (2000–2010) were extracted from reports of the Communicable Disease Surveillance in Singapore, Ministry of Health Singapore [7], [9], [26], [27], [28], [29], [30], [31], [32], [33], [34]. During the study period, a total of 4599 dengue clusters were identified with a median of 3 cases per cluster. Since 2004, dengue clusters of 10 or more cases have constituted about 7% of the total reported. In this study, we examined the time taken for vector control and cluster management in 301 (82%) of the total 368 dengue clusters which consisted of 10 cases or more (Table 1).

**Table 1:** Number of dengue clusters and clusters> = 10 cases in years 2000–2010 [34].

	2000	2001	2002	2003	2004	2005	2006	2007	2008	2009	2010	total
**1<cluster<10**	8	78	43	142	525	1097	153	891	542	375	377	4231
**cluster> = 10 cases**	1	15*	30*	38	34	93α	19	58	34	17	29	368
**Total dengue clusters**	9	93	73	180	559	1190	172	949	576	392	406	4599

* clusters not reported: 5 in 2001 & 1 in 2002;

α only clusters > = 20 cases were reported in 2005.

A dengue cluster is defined as at least two epidemiologically linked dengue cases occurring in a residential area, work place, or school within a radius of 150 m and within 14 days of onset of dengue fever. Duration of dengue cluster management equates to the length of time between identification and complete closure of a cluster. The National Environment Agency (NEA) of Singapore identified and monitored dengue clusters daily using geographical information system (GIS). Upon identification of a cluster, information was disseminated from the NEA head office via a daily dengue report to the respective regional field offices for immediate operational response. Each dengue cluster is managed by a vector control team which carries out intensive source reduction, epidemiological investigation, community engagement and surveillance until closure of the dengue cluster. If no new case is reported within 14 days from the last fever onset date, the cluster would be tentatively closed but continue to be monitored and surveillance for an additional 21 days to detect and manage any recurrence of disease.

### Statistical analysis

We identified the optimal time for dengue early warning by analyzing 1) lead time based on the risk of increasing dengue cases in each lag time between dengue and weather predictors and 2) the time frame required by local authorities to mitigate the risk of dengue outbreak using retrospective data on duration of vector control in dengue clusters.

First, we developed a Poisson regression model to analyze the relative risks of dengue cases as functions of mean temperature and cumulative rainfall at lag times of 4–20 weeks. We determined the lag times between weather predictors and dengue cases based on cross correlation function (CCF) and literature review on the effects of weather on vectors and dengue transmission [17], [18], [19], [20], [21]. Current number of dengue cases could be influenced by the number of cases in the past. We analyzed the period of this influence (autoregressive terms) using autocorrelation function (ACF), partial autocorrelation function (PACF), and literatures on dengue disease transmission [1], [35]. We applied cubic spline function on temperature and rainfall to allow a non-linear exposure and response association between weather predictors and dengue cases. We included smoothing spline of time trend from week 1 of 2000 to week 52 of 2010 with 11 degrees of freedom (*df*) to account for other influences such as circulating dominating dengue virus serotypes and vector control measures that could potentially confound the relationship between weather predictors and weekly dengue cases. Sensitivity of degrees of freedom on the effects of the smoothing function of time trend was tested with *df* range from 7–16. Furthermore, we offset midyear population to consider annual population movement and used Quasi-Poisson regression to allow for over-dispersion of data. We also estimated incidence rate ratio (IRR) between weather predictors and dengue cases using piecewise linear spline function. A best fit model was selected and validated using Akaike's Information Criterion (AIC), Generalized Cross-Validation (GCV) score, post estimation PACF, residuals diagnosis including normality and sequence plots. Data inferences were based on 95% confidence interval using R [36] and STATA 11 (StataCorp., Texas, USA).

Model:
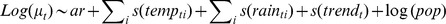

*μ_t_ represents predicted mean dengue cases in week; ar denotes autoregressive terms (2–6 weeks) of dengue cases; s(temp) denotes cubic spline of mean temperature with 3 df; s(rain) represents cubic spline of cumulative rainfall with 3 df; t = week; i = lag times of 4, 8, 12, 16, and 20 weeks; s(trend) means smoothing spline of time trend in weeks with 11 degrees of freedom; pop = midyear population*

Next, we examined the distribution of cases in identified dengue clusters, percentage of cluster-associated dengue cases, and the duration of dengue cluster management or vector control operations corresponding to the number of dengue cases in each cluster. We used the findings as indicators of estimated time or duration required for a successful mitigation. Finally, we evaluated the time gaps between possible forecast windows (lag times) and estimated time required for mitigation. Forecast-mitigation deficit or surplus was defined as the negative or positive time difference between forecast window and duration for mitigation.

## Results

### Risk of increasing dengue cases at various lag times

**Figure 1:**
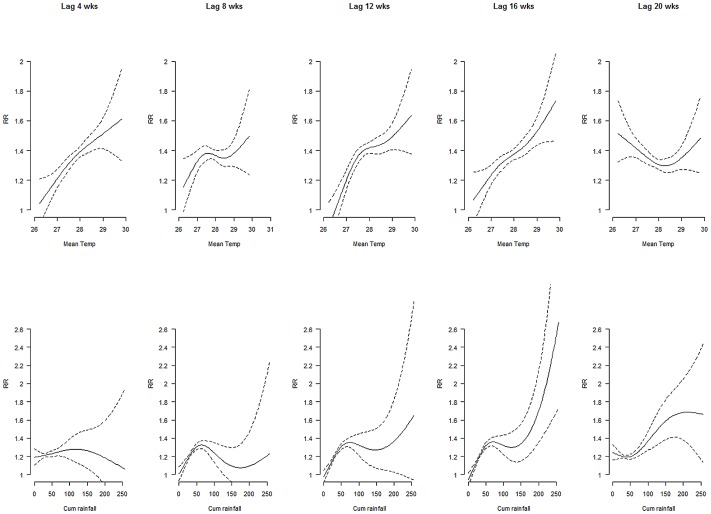
Effects of mean temperature and rain on dengue cases at various lag times. Upper panel shows relative risks of dengue cases as functions of weekly mean temperature and lower panel shows relative risks of dengue cases as functions of weekly cumulative rainfall at lag times of 4–20 weeks. Solid lines represent relative risks of dengue cases and dotted lines depict the upper and lower limits of 95% confidence intervals.

**Figure 2:**
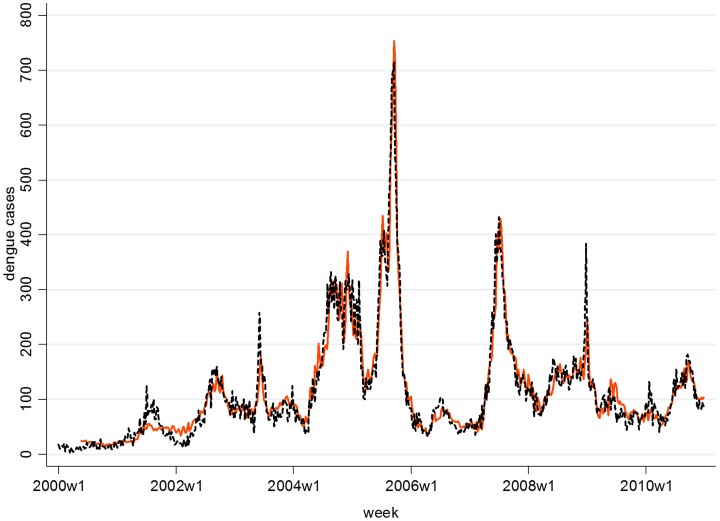
Weekly observed and predicted dengue cases from 2000–2010. Dashed line represents observed dengue cases and solid line represents predicted cases. Y-axis shows dengue cases and X-axis denotes time in week from week 1 in the year 2000 to week 52 in 2010.

 Our findings show that increasing weekly mean temperatures and cumulative rainfall precede risks of increasing dengue cases by 4–20 and 8–20 weeks, respectively (Figure 1). Each degree increase of mean temperature from 25.5°C–28.2°C elevated risk of dengue almost linearly at lag week 4–16 with peak at lag week 12; while an inverse relationship was observed between mean temperature and dengue cases at lag week 20. Figure 1 also shows mean temperature above 28.2°C raised the risk of dengue cases at lag weeks 4–20 with highest risk at lag week 16. Overall, the highest risk of dengue as a function of mean temperature occurred at lag week 12 followed by week 16. Simultaneously, each unit increase of weekly cumulative rainfall below 60 mm and above 150 mm elevated the risk of dengue cases at lag weeks 8–16 and weeks 12–20, respectively. Likewise, Table 2 shows each unit increase of weekly mean temperature raises higher incidence rate ratios for dengue cases at lag week 12 (IRR = 1.46) and week 16 (IRR = 1.39). Overall rate ratios for dengue cases in response to one mm rise of weekly cumulative rainfall peaked at lag week 16 (IRR = 1.011) and every unit increase of cumulative rainfall below 60 mm and above 150 mm elevated risks of dengue cases by 0.6% and 0.8%, respectively. Our model explained about 91% of the variance in dengue cases using weather predictors, time trend, and past cases. Residuals diagnoses and PACF indicated that the model was fit for analysis with predicted cases against observed cases as shown in Figure 2. Sensitivity tests using various degrees of freedom on the spline function of trend showed little change in risk functions.

**Table 2:** Estimated risks (IRR) of dengue cases associated with each unit increase in temperature and rainfall.

	Incidence Rate Ratio (IRR)				
	Lag 4 wks	Lag 8 wks	Lag 12 wks	Lag 16 wks	Lag 20 wks
Mean temperature (°C)					
temp<27.5	1.172	1.180	1.400	1.237	0.928
27.5 = <temp = <28.2	1.063	0.914	1.002*	1.011*	0.890
temp>28.2	1.050	0.993*	1.039	1.110	1.042
combined IRR	1.308	1.071*	1.458	1.388	0.860
95% CI (combined IRR)	1.224–1.399	0.999–1.148	1.358–1.563	1.296–1.486	0.812–0.912
Cumulative rainfall (mm)					
rain <60	1.000*	1.004	1.005	1.006	0.999*
60 = <rain = <150	0.998	0.995	0.997	0.997	1.002
rain>150	0.999*	1.002*	1.004	1.008	1.002*
combined IRR	0.998*	1.002*	1.005	1.011	1.003
95% CI (combined IRR)	0.996–1.000	0.999–1.004	1.003–1.007	1.009–1.012	1.001–1.004

* statistically insignificant wks = weeks.

### Duration of dengue cluster

**Figure 3:**
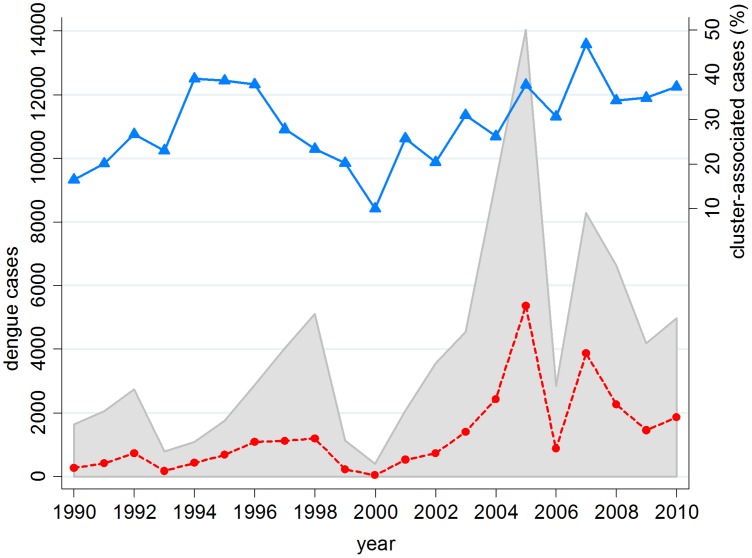
Total dengue cases, cluster-associated cases, and percentage of cluster-associated cases (1990–2010). Shaded area depicts total reported dengue cases, dotted line represents total cluster-associated dengue cases, and connected solid line with triangle markers shows the percentage of cluster-associated dengue cases over total reported cases [34].

**Figure 4:**
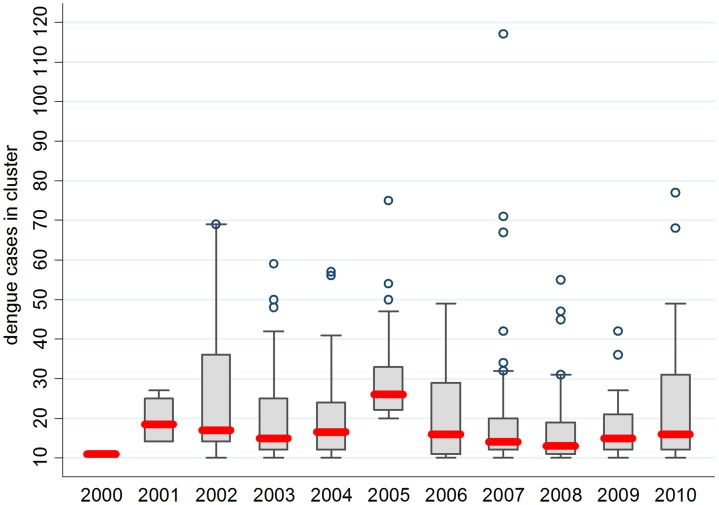
Distribution of dengue cases in clusters > = 10 cases from 2000–2010. Bold solid red line in each box indicates median number of cases in each respective year. Epidemic years = 2004, 2005, and 2007.

**Figure 5:**
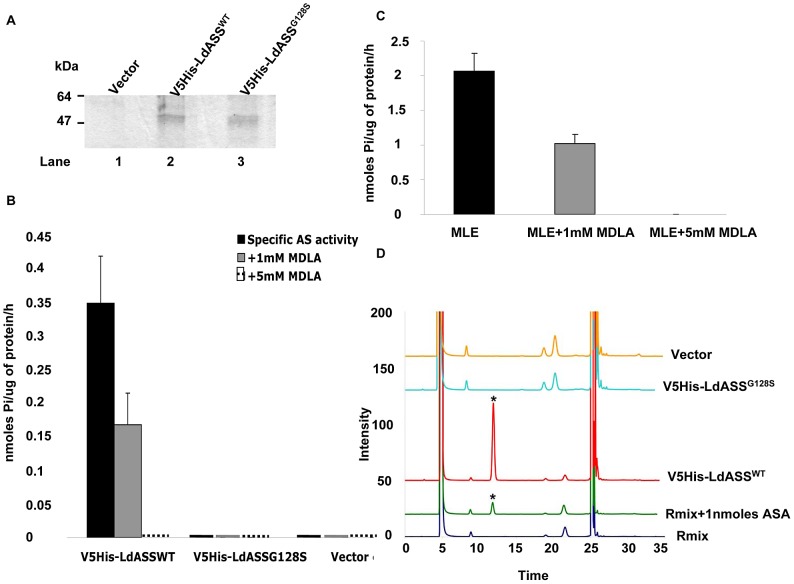
Duration of dengue clusters > = 10 cases with corresponding number of cases (2000–2010). X-axis represents strata of cluster-associated dengue cases and y-axis shows estimated time for cluster duration. Labels on y-axis denote: 1 = 1 month and less, 2 = exceeding 1 month with maximum 2 months, 3 = more than 2 months with maximum 3 months. Each bar in both panels represents percent of dengue clusters> = 10 that require corresponding duration (y-axis) for cluster management given strata of dengue cases (x-axis) in respective clusters. Left panel shows percentage distribution of dengue clusters> = 10 corresponding to cluster duration and cases in non-epidemic years. Right panel shows percentage distribution of dengue clusters> = 10 corresponding to cluster duration and cases in epidemic years (2004, 2005, and 2007).

In the past two decades, the total number of cluster-associated dengue cases contributed an average 27% of overall reported dengue cases for 1990–1999 and 30% for 2000–2010 (Figure 3). Since 2000, the proportion of cluster-associated dengue cases had been on an upward trend with a peak of 47% in year 2007. Whereas, dengue clusters that reported a minimum of 10 cases represented approximately a third of the total cluster-associated cases. From 2000–2010, the mean and median numbers of cases per cluster (> = 10 cases) were 22 and 17; mean and median numbers of cases were 21 and 16 for non-epidemic years and 23 and 19 for epidemic years (2004, 2005, and 2007), respectively (Figure 4). As shown in Figure 4, all the dengue clusters of 30 or less cases fell in the 75^th^ percentile, except in years 2002 and 2005 when only 70% of clusters had fewer than this number. The differences in time required for dengue cluster control between non-epidemic and epidemic years is minor. Figure 5 indicates most of the dengue clusters have fewer than 30 cases and take up to 2 months to control dengue outbreaks in both non-epidemic and epidemic years. During the study period, approximately 23% (non-epidemic = 28%, epidemic = 16%) of the dengue clusters were managed within 1 month, 64% (non-epidemic = 60%, epidemic = 71%) was managed within 2 months, and 13% (non-epidemic = 13%, epidemic = 13%) required maximum 3 months of vector control and cluster management to curb outbreaks (Figure 5). Longer duration (2–3 months) for cluster management was required as the number of dengue cases in each cluster exceeded 36 and 30 for non-epidemic and epidemic years, respectively. Overall, the mean and median cluster duration was about 2 months for both non-epidemic and epidemic years.

The usefulness of a dengue early warning may be reflected in the time between a forecast window and the time required by local authority to mitigate an outbreak. A forecast-mitigation deficit occurs when the duration for successful mitigation exceeds the forecast window. In view of the maximum period required to curb transmission, a 3 month forecast is deemed appropriate. Incidentally, the highest risk of dengue as a function of mean temperature and rainfall occurs at lag week 12–16. Forecasting on the basis of the observed mean temperature and cumulative rainfall 12 to 16 weeks previously could therefore provide accurate warnings while allowing sufficient time for local authorities to mitigate, or even avoid, an impending outbreak.

## Discussion

An early dengue warning system that allows time for successful mitigation will enhance effectiveness of preventive measures. Our findings show that a rise in weekly mean temperature and rainfall precede risks of increasing dengue cases by 1 to 5 months with higher risks being evident at 3–4 months. The lag times could partly be explained by high desiccation resistance of *Aedes* mosquito's eggs which could survive several months without water [37]. Our results are consistent with studies in Singapore that analyzed relationship between weekly temperature and dengue cases up to 20 weeks [4], [38]. A study in Bangkok shows that temperature and rainfall precede dengue cases up to 6 months and 3 months, respectively [39].

Using the average duration of a dengue cluster as an indicator of the period needed for successful mitigation of transmission in a localized area, our analysis has shown that the local authority typically required an average of 2 months with maximum 3 months for effective mitigation in both non-epidemic and epidemic years. As cluster management could reflect the national situation at a local level, we suggest that a similar period is required for vector source reduction to prevent an outbreak island wide. The limitations of this assumption are: 1) the time required for vector control in respond to an early warning might be shorter compare to the time needed to control vectors during an outbreak as was measured here; 2) the manpower resources allocated per unit area for an island wide source reduction effort may be smaller than those committed to local outbreak control. Nevertheless, considering that the two limitations could marginally reduce and increase the period needed for island wide preventive measures respectively, an estimation of 3 months is considered a reasonable period required for planning and implementing preventive measures. Moreover, the time lag between onset of dengue fever among cases and the identification of a dengue cluster could be a crucial but as yet unmeasured factor in outbreak prevention. Therefore, if a dengue early warning was in place, it is possible that the maximum mitigation duration could be even less than 3 months.

A dengue early warning at the optimal time boosts the success of vector control operations and cost-effectiveness of intervention. A study by Oki *et al.* (2011) has suggested that optimal timing of a vector control such as insecticide fogging increases the impacts of intervention on reduction of dengue cases [40]. Likewise, optimal timing of an early warning of dengue outbreak inevitably increases the impact of the early warning on the effectiveness of preventive measures. Dengue control programs are an economic burden to government and communities. During 2000 to 2009, dengue control in Singapore cost approximately US $500 million to the nation [41]. Effective vector controls could help to minimize the economic burden possibly by reducing the number of dengue cases, preventing loss of working days and income due to disease, increasing saving on disability-adjusted life years (DALY) and boosting effectiveness of each dollar spent on intervention [42].

The duration of dengue cluster management could be influenced by a complex spatiotemporal interplay of risk factors unique to respective dengue clusters: 1) Demographic characteristics, density, and herd immunity among host population or residents in clusters could have an influence on the number of dengue cases. A community with lower herd immunity could possibly experience dengue epidemic with a low mosquito-population density [3], [43], 2) Environmental factors such as conditions, types and ages of structural buildings, construction activities, public drainage systems, and presence of parks possibly increase the challenges and prolong the duration of vector control operations in certain clusters, 3) Differential vector populations due to inaccessibility of larval breeding sites, larval indices, density of adult *Aedes aegypti*, and numbers of potential breeding sites complicate the dynamics of dengue transmission and require greater effort for outbreak control in respective clusters, 4) With the co-circulation of all four serotypes of dengue virus in Singapore, the circulating serotypes and herd immunity to the specific serotype of concern also influence the number of cases in clusters, 5) Community commitment to prevent dengue has a direct impact on the duration of vector control. Residents not granting permission to dengue officers to enter their premises to conduct mosquito larvae inspection and elimination could prolong the duration as well as reduce effectiveness of vector control measures.

Since 2005, Singapore has monitored circulating dengue virus to detect switches in predominant serotype which could signal an impending outbreak. More recently, virus genetic data has shown that clade replacement without a switch in serotype, could also lead to increase in cases [44], [45]. Here we determine that 3 months lead time could be optimal for a warning to be issued; and that temperature and rainfall data could provide a forecast in that timeframe, to allow for preparation of control measures. Intervention measures include public and stakeholders' engagement, gaining political support and systematic source reduction exercises. The findings in this study were geographically based because of the heterogeneity of the environment including local weather, circulating viral serotypes, and herd immunity in the respective study areas. Owing to the fact that a model-based dengue early warning system has not previously been adopted for dengue surveillance in the study area, we utilized existing available vector control and cluster duration data as indicative references. Further studies could be undertaken to evaluate the forecast-mitigation gaps more accurately when such data are available in the future.

Our study identified a short term forecast window of 3–4 months. In an environment where long duration of response or mitigation is anticipated, the effectiveness of a dengue early warning can be improved by re-considering the forecast-mitigation gap. One approach is to forecast dengue outbreaks with a longer lead time using other predictors of weather. Several studies in various geographical areas have revealed the feasibility of forecasting dengue cases several months in advance using weekly or monthly weather predictors and up to 10 months ahead using Southern Oscillation Indices (SOI), El Niño Index, or El Niño Southern Oscillation indices (ENSO) [23], [46], [47], [48]. Although long term forecast could possibly be compromised by lower forecast precision, the long lead time may be useful for longer term planning such as allocation of resources and acquisition of control tools such as insecticides.

For several decades, dengue control has been a challenge for regions where dengue is endemic. Optimal timing of an early warning could help to bridge the forecast-mitigation gap between theoretical research predictions and practical control operations. Identifying the optimal lead time for dengue forecast and duration of local vector control could help to improve the functional aspects of a dengue forecasting model, reduce risk of dengue epidemic, increase cost-effectiveness of control strategies, and encourage local authorities to adopt a model-based dengue early warning. The lead time needed for mitigation varies according to different influencing factors in respective study areas. We emphasize the importance of considering the forecast-mitigation gaps in respective study areas when developing a dengue forecasting model.
